# Frequency of Thyroid Microcarcinoma in Patients Who Underwent Total Thyroidectomy with Benign Indication—A 5-Year Retrospective Review

**DOI:** 10.3390/medicina60030468

**Published:** 2024-03-12

**Authors:** Vasiliki Magra, Kassiani Boulogeorgou, Eleni Paschou, Christina Sevva, Vasiliki Manaki, Ioanna Mpotani, Stylianos Mantalovas, Styliani Laskou, Isaak Kesisoglou, Triantafyllia Koletsa, Konstantinos Sapalidis

**Affiliations:** 1 Third Department of General Surgery, Medical School, AHEPA University Hospital, Aristotle University of Thessaloniki, 54621 Thessaloniki, Greece; paschoueleni@gmail.com (E.P.); christina.sevva@gmail.com (C.S.); steliosmantalobas@yahoo.gr (S.M.); ikesis@hotmail.com (I.K.); sapalidiskonstantinos@gmail.com (K.S.); 2 Department of Pathology, Faculty of Medicine, School of Health Sciences, University Campus, Aristotle University of Thessaloniki, 54124 Thessaloniki, Greece; siliaboulog@gmail.com (K.B.); tkoletsa@auth.gr (T.K.); 3 School of Medicine, Aristotle University of Thessaloniki, 54124 Thessaloniki, Greece; vassiamanaki@gmail.com (V.M.); ioannampo@hotmail.co.uk (I.M.)

**Keywords:** thyroidectomy, papillary, microcarcinoma, incidental, thyroid

## Abstract

*Background and Objectives*: Incidental thyroid cancers (ITCs) are often microcarcinomas. The most frequent histologic type is a papillary microcarcinoma. Papillary thyroid microcarcinomas are defined as papillary thyroid tumours measuring less than 10 mm at their greatest diameter. They are clinically occult and frequently diagnosed incidentally in histopathology reports after a thyroidectomy. The aim of this study is to evaluate the rate of papillary thyroid microcarcinomas (PTMC) in patients who were thyroidectomised with indications of benign disease. *Materials and Methods*: We retrospectively evaluated the histological incidence of PTMC in 431 consecutive patients who, in a 5 year period, underwent a thyroidectomy with benign indications. Patients with benign histology and with known or suspected malignancy were excluded. *Results*: Histopathology reports from 540 patients who underwent a total thyroidectomy in our department between 2016 and 2021 were reviewed. A total of 431 patients were thyroidectomised for presumed benign thyroid disease. A total of 395 patients had confirmed benign thyroid disease in the final histopathology, while 36 patients had incidental malignant lesions (33 PTMC—7.67%, one multifocal PTC without microcarcinomas—0.23%, two follicular thyroid carcinoma—0.46%). Out of the PTMC patients, 29 were female and four were male (7.2:1 female–male ratio). The mean age was 54.2 years old. A total of 24 out of 33 patients had multifocal lesions, 11 of which co-existed with macro PTC. Nine patients had unifocal lesions. A total of 21 of these patients were initially operated on for multinodular goitre (64%), while 13 were operated on for Hashimoto/Lymphocytic thyroiditis (36%). *Conclusions*: PTMC—often multifocal—is not an uncommon, incidental finding after thyroidectomy for benign thyroid lesions (7.67% in our series) and often co-exists with other incidental malignant lesions (8.35% in our series). The possibility of an underlying papillary microcarcinoma should be taken into account in the management of patients with benign—especially nodular—thyroid disease, and total thyroidectomy should be considered.

## 1. Introduction

Thyroid cancer is the most frequent type of endocrine cancer. A papillary thyroid carcinoma is the most frequently encountered thyroid tumour and comprises 70–90% of well-differentiated thyroid cancers [1]. Incidentally found thyroid carcinomas imply lesions that were not clinically suspected prior to their diagnosis. In previous years, these carcinomas were all considered incidentalomas and were not specifically categorised. More recently, they were thought of as a separate entity and were classified into four broad categories based on the way they are diagnosed [2]: (i) microcarcinomas diagnosed incidentally in histopathology reports post thyroidectomy despite no clinical suspicion for malignancy pre-operatively, (ii) microcarcinomas incidentally detected during imaging, usually ultrasound, which were confirmed with fine needle aspiration cytology (FNAC), (iii) carcinomas that first present with lymph node metastases and the primary lesion—a microcarcinoma—is identified in post-operative histopathology, and (iv) carcinomas in ectopic thyroid tissue that present with metastases or clinical symptomatology [2]. In clinical practice, these microcarcinomas are most frequently found in histopathology reports of thyroidectomy specimens in patients with benign indications for surgery [3]. The most frequent histotype encountered is a papillary thyroid microcarcinoma (PTMC).

The World Health Organisation (WHO) defines thyroid microcarcinoma as thyroid carcinoma with a maximum diameter under 1 cm and without including any further tumour characteristics, such as the presence of metastatic lesions or local extra-thyroidal extension of the tumour [4]. The recently published WHO classification of thyroid tumours (2022) notes that a papillary thyroid carcinoma should be classified based on the tumour’s histopathologic characteristics, irrespective of size. It is also stressed that tumours under 1 cm in diameter should not be considered low-risk simply because of their size [5]. Papillary thyroid microcarcinomas are usually multifocal and most frequently found in thyroid glands with a multinodular pathology.

The improved sensitivity of imaging modalities, especially ultrasound, as well as the increased accuracy of histopathology examination of the thyroid specimen, can explain the increased incidence of incidentally found thyroid microcarcinomas [1]. The incidence of papillary thyroid microcarcinomas in autopsy and clinical studies is 100- to 1000-fold higher than the incidence of such tumours that develop into clinically apparent cancers encountered in clinical practice [2]. It is, therefore, reasonable to consider if the diagnosis of a papillary microcarcinoma should automatically become an indication for surgical resection.

The clinical significance of papillary thyroid microcarcinomas and their clinical course remains a matter of interest and debate. Papillary thyroid microcarcinomas do not appear to be a unified entity. The clinical progress of these lesions is not yet clear or specific. While the majority have an excellent prognosis, with a 20 year mortality rate of less than 1%, they do have metastatic potential to neck lymph nodes (via lymphatic spread) and less frequently to distant sites (via haematogenous spread). It has also been observed that 4–16% of papillary thyroid microcarcinomas recur locally after initial excision, and a significant percentage of these patients develop distant metastases [6]. Roti et al. tried to identify the clinical characteristics that differentiated incidentally diagnosed papillary microcarcinomas from thyroid lesions suspicious of malignancy. The only differentiating factor between the two groups was size. No lesions smaller than 8 mm in diameter developed metastases [7].

At this point, it is important to mention a subtype of papillary thyroid microcarcinoma, an aggressive PTMC. Ardito et al. conducted a retrospective study on papillary thyroid microcarcinoma and concluded that although most of them had uncomplicated clinical progress, 19% recurred [8]. The microcarcinomas that recurred had common histopathologic characteristics. More specifically, they were multifocal, lacked a capsule, and were solid. As a result, they suggested that this subtype of papillary thyroid microcarcinomas should be treated aggressively with a total thyroidectomy, central lymph node clearance, and radioiodine [8].

There is currently no consensus regarding the clinical progression of papillary thyroid microcarcinomas or the most appropriate treatment strategy. Options range from watch and wait to a total thyroidectomy with central lymph node clearance and radioiodine, as in the patients from the aforementioned study. The American Thyroid Association 2015 guidelines suggest that for cN0 tumours < 1 cm without extrathyroidal extensions, a hemithyroidectomy should be the operation of choice [9]. Despite this guideline, many authors suggest that a total thyroidectomy and central lymph node clearance should be the treatment strategy of choice due to the frequency of multifocality of such tumours as well as their tendency to metastasise to level IV lymph nodes in the neck. This avoids re-operation and reduces the risk of local recurrence. Spaziani et al. [10] published a recent prospective observational study that identified a high percentage of incidental papillary thyroid microcarcinomas (15.3%) with high rates of bilateral lesions and multifocality (63.1%). They go on to suggest a total thyroidectomy as the surgical treatment of choice, even for presumed benign thyroid disease [10]. The main counterargument is the increased frequency of central lymph node clearance, especially permanent hypocalcemia [11].

This retrospective study aims to investigate the frequency and multifocality of incidentally diagnosed thyroid papillary microcarcinomas within the thyroid gland. The literature suggests that the incidence of such tumours ranges from 3% to 16%, although, occasionally, much higher numbers have been documented (up to 81%) [1]. Keeping in mind the high incidence of these incidental microcarcinomas, as well as their tendency for multifocality, a total thyroidectomy seems to be a reasonable treatment strategy, especially in cases of underlying multinodular goitre when both thyroid lobes are affected.

All the patients included in this study underwent a total thyroidectomy. Given that the main counterargument against a total thyroidectomy vs a hemithyroidectomy for benign thyroid lesions is the increased rate of complications, we mention the postoperative complications in the patient cohort studied and proceed to compare them to the international literature.

## 2. Materials and Methods

This is a 5 year retrospective study for which we retrieved 540 histopathology reports of patients that underwent a total thyroidectomy at the 3rd General Surgical Department of AHEPA University Hospital from 2016 until 2021. A total of 145 histopathology reports confirmed thyroid malignancies, while 395 reports confirmed benign thyroid lesions (multinodular goitre, Graves disease, and Hashimoto thyroiditis). A total of 58 papillary microcarcinomas were confirmed, 33 of which were incidental findings in patients who underwent a total thyroidectomy with benign pre-operative indication. These were divided into unifocal and multifocal; their mean diameter and the standard deviation (SD) were calculated, as well as the number of lesions in multifocal carcinomas.

Patients with a confirmed pre-operative diagnosis of malignancy (post-fine needle aspiration cytology) were excluded from the study. Patients with histopathology that confirmed exclusively benign thyroid diagnoses were also excluded from the study. Finally, 2 patients with incidentally diagnosed follicular thyroid carcinomas were excluded. All patients underwent a preoperative endocrinology assessment, clinical examination of the neck, laboratory examination for thyroid hormones (TSH, T3, T4, procalcitonin, anti-thyroid peroxidase (anti-TPO), and thyroglobulin), neck/thyroid ultrasound, pre-operative laryngoscopy, and, based on indication, a neck computed tomography (CT) scan and fine needle aspiration (FNA). Intraoperative nerve monitoring was used in all operations.

There was telephone contact with 431 patients who underwent a total thyroidectomy due to presumed benign thyroid disease to confirm complications and recurrence. The histopathology reports of all patients were also reviewed, and parathyroid glands were included. Complications were divided into immediate/transient and permanent.

## 3. Results

From a total of 540 patients, 431 had a preoperative benign indication for total thyroidectomy (395 had confirmed benign diagnosis in the histopathology report of the thyroidectomy specimen, 33 had incidentally diagnosed papillary microcarcinomas, one had incidentally diagnosed papillary macro carcinoma and two had incidentally diagnosed follicular carcinomas). In total, 33 out of 431 (7.67%) patients were found to have papillary thyroid microcarcinomas. These patients had benign indications for thyroidectomy pre-operatively and no suspicion of thyroid malignancy during the pre-operative work-up. These patients constitute the focus of this study.

Out of the 33 patients with incidentally diagnosed papillary microcarcinomas, the pre-operative diagnosis for 20 of them (60.6%) was a multinodular goitre, and for the remaining 13 patients (39.4%), Hashimoto thyroiditis. These diagnoses were confirmed in the histopathology reports post-operatively. There were no papillary microcarcinomas in the histopathology specimen of patients with Graves disease. A total of 29 patients were women, and four were men (7.2:1 F–M ratio). The mean age for women was 54.2 years old (31–72) and 54.2 years old (50–59) for men.

A total of 24 patients (72.7%) had multifocal papillary microcarcinomas (23 women with a mean age of 52.2 years old and one 52 year old man). Nine patients (27.3%) had unifocal papillary microcarcinomas (six women with a mean age of 61.8 years old and three men with a mean age of 55 years old). The mean maximal diameter of multifocal microcarcinomas was 3.5 mm ± 0.19 mm SD, and for unifocal microcarcinomas was 3.5 mm ± 0.23 mm SD. The mean number of lesions in multifocal microcarcinomas was 2.83 (2–6 lesions per patient). In 11 (45.8%) out of 24 patients, multifocal papillary microcarcinomas co-existed with papillary thyroid carcinomas (macro PTC > 1 cm).

There was telephone communication with 431 patients who underwent a total thyroidectomy with a benign indication for surgery. Contact was not achieved with eight of these patients. Patients were asked about immediate/transient and permanent post-operative complications, specifically about hypoparathyroidism and vocal cord palsy symptoms (numbness, hoarseness of voice, stridor, breathing difficulty, and emergency tracheostomy). A total of 11 out of 423 patients (2.60%) reported transient hypocalcaemia, three reported permanent hypocalcaemia (0.70%), and four reported transient unilateral vocal cord paresis (0.94%), which normalised in repeat laryngoscopy one year post-operatively. No patients reported stridor or required an emergency tracheostomy due to bilateral vocal cord paresis. Parathyroid glands were found in 5 out of 431 (1.16%) histopathology reports, four of which contained one parathyroid gland, while 1 contained two parathyroid glands. There were no cases of local recurrence, lymphatic spread, or distant metastases to date. Table 1 below shows the characteristics of the patients and the microcarcinomas, focusing on the multifocality of the tumour.

## 4. Discussion

Papillary thyroid microcarcinomas are a unique subdivision of papillary thyroid carcinomas, which heralds attention due to their increasing frequency as well as the implications for patient management [12]. Although currently considered a unified category of thyroid tumours, many authors have started to consider papillary thyroid microcarcinomas as a group of tumours that may exhibit different biological behaviours. This would explain why some microcarcinomas have an indolent course while others behave aggressively. Different studies have suggested many risk factors for the more aggressive subdivision of papillary thyroid microcarcinomas without a unified opinion in the literature.

Papillary thyroid microcarcinomas and truly incidental thyroid carcinomas do not always belong to the same category. It is possible to detect incidental microcarcinomas in patients with an already-known thyroid carcinoma. This group of microcarcinomas would not be strictly defined as incidental. In this study, 63.6% of patients with incidental thyroid papillary microcarcinomas had a preoperative diagnosis of multinodular goitre. This finding is in accordance with reports in the literature, as microcarcinomas are encountered more frequently in nodular thyroid glands. The incidental diagnosis of a PTMC in patients with multinodular goitre requires frequent monitoring. The management of such patients should be dictated by the characteristics of the lesion as well as the age and sex of the patient, which Bove et al. found to be the most decisive factor [13]. These patients should be regularly monitored due to their high risk of developing papillary thyroid carcinomas.

Multiple authors have investigated the link between Hashimoto thyroiditis and an increased risk of developing thyroid cancer, especially papillary thyroid cancer. In this study, 39.4% of the patients incidentally diagnosed with a PTMC had histopathologically confirmed Hashimoto thyroiditis (HT)/chronic lymphocytic thyroiditis (CLT). Hu et al. [14] performed a systematic review and meta-analysis of the cancer risk in Hashimoto’s thyroiditis and found an increased risk of developing thyroid cancer (RR = 1.49 *p* < 0.067). The increased risk is attributed to chronic inflammation [14,15,16,17,18], especially through the release of free oxygen radicals, inflammatory cytokines [14], and the expression of oncogenes [15,18]. Unger et al. [15] found a potentially linked expression of p63 in patients with Hashimoto’s thyroiditis and patients with papillary thyroid carcinomas. Jackson et al. [16] also found a 1.5-fold increased risk of developing PTCs in patients with chronic lymphocytic thyroiditis while conducting a retrospective study of 1268 patients. Vargas-Uricoechea [17] notes a potential pathobiological link between autoimmune thyroid disease (Graves and HT) and thyroid cancer, especially papillary thyroid carcinoma, during their review of the literature. They stress, however, that the existing evidence from observational studies is not enough to establish causality [17]. Interestingly, they mention that the category of patients with “indeterminate” nodules (Bethesda III and IV) may benefit from better classification by using molecular markers [17]. Finally, Lee et al. [18] conducted a 9 year retrospective study of 2928 patients and found that chronic lymphocytic thyroiditis correlated positively with multifocality, smaller tumour size, extrathyroidal extension, and p53 expression but negatively with lymph node metastases.

Due to the uncertain clinical progression of papillary thyroid microcarcinomas, communication with the patient is key so that they are fully informed about the risks and benefits of different management options—surgical vs. active monitoring—and that they can be actively involved in the decision-making process [19]. Fine needle aspiration is known to be useful in the diagnosis of thyroid nodules. However, the role of fine needle aspiration cytology (FNAC) in patients with multinodular goitre remains controversial, especially when aiming to diagnose microcarcinomas. Lasithiotakis et al. reported suspicious FNA findings for carcinomas in 6.7% of cases [19], while Baier et al. had a 12% rate of false negative samples [20]. Miccoli et al. found the role of fine needle aspiration cytology in their study of incidental thyroid carcinomas to be inconclusive, even for tumours larger than 2 cm [21]. Evranos et al. concluded that cytologically suspicious nodules proved to be benign in surgical pathology, while cancer foci were identified in previously undefined areas of the thyroid in patients with incidentally diagnosed carcinomas [22]. None of the 33 patients that were included in this study had suspicious features on imaging and thus did not undergo FNA.

The size of microcarcinomas poses challenges in diagnosis both when imaging and when performing fine needle aspiration on such lesions. In the current study, the mean maximal diameter of multifocal PTMC and unifocal PTMC was 3.5 mm ± 0.19 SD and 3.5 mm ± 0.23 SD, respectively. A total of 11 patients had co-existing papillary thyroid carcinomas > 1 cm in diameter. It is evident that in cases of underlying multinodular goitre, the challenges are increased due to the morphology of the underlying gland. Lucandri et al. support this as they found that the mean diameter of non-incidental papillary thyroid microcarcinomas is double when compared to the incidental PTMC group (7.2 vs. 3.8 mm; *p* < 0.001) [23].

In most cases of patients with papillary thyroid microcarcinoma, the life expectancy is excellent, with a mortality of <1% in 20 years [1]. Despite the excellent life expectancy, papillary thyroid microcarcinomas often metastasise to cervical lymph nodes and occasionally to distant sites [1]. In the 4–16% of patients that have a local recurrence, the risk for distant metastasis is significantly higher [1]. Kaliszewski et al. [24] investigated the high-risk characteristics of papillary thyroid microcarcinomas in order to aid in guiding the management of such patients, especially when considering active surveillance versus more limited surgical resections, for example, a hemithyroidectomy. They found that an age of ≥55 years, hypoechogenicity, microcalcifications, irregular tumour shape, smooth margins, and high vascularity increased the risk for minimal extrathyroidal extension, lymph node metastasis, and capsular and vascular invasion [24]. It is certainly evident that some of these characteristics are very difficult to identify, both due to the small size of these lesions as well as due to the frequent lack of clinical suspicion. The findings of Slijepcevic et al. [25] are in agreement with Kaliszewski et al. [26] when it comes to the size of the lesion. They both mention that lesions under 5 mm are less likely to have intrathyroid extension and lymph node or distant metastases. The characteristics of imaging, and especially conventional ultrasound that is mentioned above (hypoechogenicity, microcalcifications, tumour shape, margins, and vascularity), are useful when aiming to categorise thyroid nodules, especially when attempting to identify malignant nodules. The Thyroid Imaging Reporting and Data System (TIRADS) has been developed and used widely based on such ultrasound characteristics. The TIRADS system is not particularly useful in “indeterminate nodules”, for example, TIRADS III. Chen et al. [27] used conventional ultrasound-based radiomics to create a predictive model for identifying papillary thyroid microcarcinomas in patients with TIRADS III nodules. They created three predictive models: one based on radiomics, one using clinical features, and a predictive model combining both. They found excellent predictive results for papillary thyroid microcarcinoma in TIRADS III nodules. Interestingly, there was no statistical difference between the radiomics-based model and the combined radiomics-clinical-based model [27]. These results show promise for more accurate diagnostic techniques and better risk stratification of patients with benign thyroid disease and suspected papillary thyroid microcarcinomas.

In recent years, molecular profiling has become more feasible due to next-generation sequencing technology. Molecular profiling is the focus of many studies for different types of cancer. There is hope that these studies will aid the overall individualisation of risk profiling and cancer management in the future. Li et al. [28] performed whole exome and RNA sequencing on 64 patients with a papillary thyroid microcarcinoma (PTMC) and compared their findings to the patients in the Cancer Genome Atlas Program (TCGA). Apart from the well-known BRAF and RET mutations, they identified a molecular signature that they named PTMC-inflammatory, which they hypothesise presents a link to immune intervention [28]. Molecular profiling and further understanding of the immune microenvironment of these tumours can aid in the better classification of thyroid papillary microcarcinomas and risk stratification of patients. It can also provide potential targets for individualised treatment strategies, especially immunotherapy.

Papillary thyroid microcarcinomas are often multifocal (15.5–40% in surgical series and >80% in systematic autopsy studies) [1]. In the current study, 72.7% of PTMCs were multifocal, a percentage higher than reported in most surgical series. Kaliszewski et al. showed that multifocal or bilateral tumours and tumour sizes above 5 mm were the best predictors of lymph node metastasis in papillary thyroid microcarcinomas and thus require a more aggressive treatment approach; capsular invasion, age, and gender were not found to be statistically significant risk factors [26]. They also note that in cases of lymph node metastases or tumours larger than 5 mm, the entirety of thyroid tissue should be pathologically examined to exclude the multifocality of PTMCs [26]. This would result in a completion thyroidectomy in case a thyroid lobectomy was initially performed. Maturo et al. noted that the microcarcinoma foci were frequently found in the Zuckerkandl tubercle or in the pyramidal lobe, areas that are often not excised during thyroid lobectomy [29].

One of the main arguments used against a total thyroidectomy in benign thyroid disease is the increased risk for postoperative hypoparathyroidism as well as injury to recurrent laryngeal nerves. The postoperative complication rates reported in this study, 0.70% hypoparathyroidism and 0.94% unilateral vocal cord paresis, are similar to the postoperative complication rates reported in patients who underwent lobectomy [29,30]. Studies from specialist thyroid surgery units argue that the recurrent laryngeal nerve dissection technique, as well as thyroid vessel ligation near the thyroid capsule in total thyroidectomy by specialist thyroid surgeons, reduces the complication rates to levels comparable to lobectomy [29,30,31]. It needs to be stressed that a completion thyroidectomy in case of local recurrence post-lobectomy is certainly a more challenging operation with much higher complication rates [30].

The main limitation of this study is that it is a retrospective study from a single centre. The main benefit of the study, apart from the reasonable number of participants, is that all patients were operated by the same specialist endocrine surgical team and all specimens were examined in the same histopathology laboratory by histopathologists specialising in endocrine and thyroid pathology.

## 5. Conclusions

Papillary thyroid microcarcinomas are a common incidental finding in patients with benign thyroid disease, especially multinodular goitre. The incidental diagnosis of such lesions presents a challenge for the endocrine surgeon and can be especially stressful for the patient. It is important to consider all therapeutic options and tailor them to each patient based on their individual risk profiles in addition to the imaging characteristics and cytological characteristics of the microcarcinoma.

Based on the findings of this study, as well as a review of the literature, a total thyroidectomy should be considered as the operation of choice in patients with benign thyroid disease requiring surgical excision, especially in the case of multinodular goitre. Patient selection as well as open communication with the patient and the multidisciplinary team remain key in the management and follow-up of these patients.

There are multiple studies focusing on novel techniques for risk stratification and diagnosis of patients with papillary thyroid microcarcinomas. The improvement of imaging techniques, as well as the incorporation of computational methods and the development of radiomics, provide promise for more accurate diagnosis and individualisation of treatment approaches for patients with thyroid microcarcinoma. It should aid in providing the utmost diagnostic accuracy in order to avoid overtreatment without misdiagnosing in this particular patient group. Further prospective, randomised trials, as well as studies focusing on the molecular characteristics and immune microenvironment of papillary thyroid microcarcinomas, are needed in order to understand this distinct category of thyroid tumour and to determine the best treatment strategy for this increasingly common and diverse group of patients.

## Figures and Tables

**Table 1:** Characteristics of patients with papillary thyroid microcarcinoma.

Characteristics	Female	Male	Total
Multifocal tumours	23	1	24
Unifocal tumours	6	3	9
Mean age	54.2 years old (31–72)	54.2 years old (50–59)	n/a
Multinodular goitre	16	4	20
Hashimoto thyroiditis	13	0	13

## Data Availability

Data are contained within the article.
